# Altered Phenotype and Gene Expression of Regulatory T cells (Tregs) in Children with Autism, and the Relationship with Comorbid Gastrointestinal Symptoms

**DOI:** 10.21203/rs.3.rs-8080761/v1

**Published:** 2025-11-18

**Authors:** Rachel J Moreno, Destanie Rose, Paul Ashwood

**Affiliations:** UC Davis; UC Davis; UC Davis

**Keywords:** autism, immune, regulatory T cells, Tregs, inflammation, behavior, gut, GI, metabolism, gene expression

## Abstract

Autism Spectrum Disorder (ASD) is a neurodevelopmental condition characterized by social deficits and stereotypic behaviors. Increased numbers of inflammatory cells and their mediators have been found in peripheral blood, brain, and gastrointestinal (GI) tissues of individuals with ASD. Regulatory T cells (Tregs) play a crucial role in suppressing inflammatory processes that, if disrupted, can result in inflammation and development of a variety of immunological conditions. In this study, we sought to characterize Tregs populations using flow cytometry and gene transcriptomic approaches in children with ASD (n = 36) and typically developing (TD) children (n = 18) enrolled in the Childhood Autism Risk from Genetics and Environment (CHARGE) study. We also examined differences in the frequencies of activated Tregs in ASD groups when stratified by co-occurring GI status. The frequency of gut homing α4b7^+^Tregs positive for inhibitory receptors GITR, LAP, and/or GARP were altered based on the presence of GI symptoms. Analysis of mRNA isolated from CD4^+^CD25^+^Tregs, showed 213 differentially expressed genes (DEGs) between the ASD and TD children. Upregulated DEGs were enriched in Gene Ontology (GO) Biological Processes involved in epigenetic regulation including ‘chromatin organization’, whereas Kyoto Encyclopedia of Genes and Genomes (KEGG) pathways were involved in metabolism, including ‘lipid and atherosclerosis’ and ‘pantothenate and CoA biosynthesis’. Upregulation of immune signaling genes (MAPK3, JAK2, and CASP3) was also noted in the ASD Tregs. Downregulated DEGs consisted of genes enriched in immune terms including the GO term ‘leukocyte differentiation’ and KEGG pathways ‘Parkinson disease’ and ‘protein processing in endoplasmic reticulum’. Correlation analysis revealed a relationship between inappropriate speech scores and lower frequencies of Tregs in ASD children. Overall, these data support the hypothesis of altered Tregs cell biology in ASD, including their lower frequencies and altered gene expression. Furthermore, altered metabolic and immune signaling processes may contribute to changes in frequencies of Tregs in ASD based on co-morbidities, ultimately driving the immune status in ASD from a balanced to a dysregulated/inflammatory state.

## Introduction

Autism spectrum disorder (ASD) is a behavioral disorder characterized by restrictive and repetitive behaviors, as well as communication and social deficits [1]. The prevalence of ASD continues to rise from what used to be a rare diagnosis 20 years ago to nearly 3% of the population, affecting 1 in 31 children in the US [1]. Some of these changes may be the result of diagnostic criteria expanding over time and including previously understudied groups. Causes of ASD have been attributed to genetic pre-dispositions, increased environmental exposures to noxious agents, and the transmission of an altered epigenome throughout generations. Many of the environmental and genetic factors considered to contribute to ASD etiology are also involved in regulating the immune system [2]. For example, genes that are related to immune activation (e.g. mTOR and PTEN) are commonly implicated in syndromic ASD cases [3, 4]. Moreover, environmental exposures early in life, such as stress, maternal asthma, exposure to gestational infections, or maternal dysbiosis have also been found to impact the immune system [5–7]. Aberrant immune activity or inflammation is also frequently observed in individuals with ASD, with strong evidence suggesting that the severity/degree of immune activation is associated with increased impairments in behavior [8]. Heightened immune activity may leave individuals with ASD more at risk of developing immune mediated comorbidities such as autoimmunity, allergies and gastrointestinal (GI) issues. This is exemplified by the fact that ASD individuals are 6 to 8 times more likely of developing GI issues, or an increased prevalence of autoimmune conditions such as psoriasis [9, 10].

Immune dysregulation, particularly T cell dysfunction, is central to many of the co-morbidities that children with ASD are at risk of developing [11]. Increased lymphocyte activation, proliferation, effector programs and proinflammatory cytokines have been observed in children with ASD compared to typically developing (TD) children [12–14]. Moreover, previous studies from our group suggests that T cell phenotypes in children with ASD can differ depending on whether GI issues are present. Children with ASD with GI issues had elevated T helper (T_H_)17 and T_H_17.1 populations, whereas children with ASD without GI symptoms had increased frequencies of T_H_2 populations [15]. We also found that children with ASD with or GI issues have differing cytokine responses after peripheral lymphocyte stimulation, and that the degree of the response is related to more impaired behavioral outcomes compared to children with ASD without GI issues [13, 16]. Immune dysregulation, however, is not restricted to T cells, as many cells of the innate immune system are more pro-inflammatory in ASD in general and altered in those with GI issues [17, 18]. Collectively, this data implies that immune activation occurs in children with ASD and may drive different outcomes in ASD individuals such as co-morbidfeatures.

Regulatory T cells (Tregs) are essential for maintaining immune tolerance against self-proteins, controlling the immune response and limiting collateral damage following infections [19]. Tregs regulate immune activity via soluble factors and through cell-contact dependent inhibitory mechanisms. Tregs secrete anti-inflammatory cytokines such as transforming growth factor (TGF)β1, interleukin (IL)-10 and IL-35 that have broad effects on the local environment, including downregulation of co-stimulatory receptors and inflammatory cytokine production in innate cells and inhibiting T effector cell programs and T effector cell activation. Reduced plasma levels of anti-inflammatory TGFβ1 and IL-35 in children with ASD have previously been associated with worse scores on the aberrant behavior checklist (ABC) assessment [20, 21]. In the gut, reduced frequencies of IL-10^+^CD3^+^ T cells were observed in ASD and associated with increased mucosal inflammation [22]. Furthermore, there are a variety of cell-contact dependent inhibitory receptors on Tregs that engage to suppress the immune responses, such as CTLA-4, which inhibits T effector cell stimulation by competing for binding of CD80/CD86 on antigen presenting cells. Lower frequencies of Tregs have been reported in the peripheral blood of ASD children [23–26]. However, the exact Tregs phenotypes reported varied between studies. Forkhead box protein P3 (Foxp3) is a transcription factor whose expression is enriched in Tregs. In ASD, Foxp3^+^ expression is altered, and polymorphisms in the Foxp3 promoter and miRNAs that regulate its expression have been described [27, 28]. Cell surface marker IL-2 receptor a chain (CD25^+^) is also associated with Tregs populations. In children with ASD, CD25 fails to be upregulated upon stimulation and frequencies of CD4^+^CD25^+^ cells are decreased [11]. Collectively, these data suggest there may be a dysregulation in Tregs biology in ASD that could contribute to increased inflammation and more behavioral impairments. Despite their apparent role in ASD pathology, Tregs have not been extensively studied in ASD. Characterization of this regulatory population can provide important insights into the inflammatory processes that occur in ASD. In this study, we examined Tregs isolated from the peripheral blood of children with ASD enrolled in the Childhood Autism Risk from Genetics and Environment (CHARGE) study, analyzing Tregs phenotype using flow cytometric and high throughput gene expression sequencing methods. We also stratify our analysis into ASD groups with and without GI issues to identify differences driving or potentially driven by the presence of gastrointestinal co-morbidities.

## Methods

### Subject Information

Children enrolled in the CHARGE study were grouped in either ASD (n = 36, 8 female) and typically developing (TD) (n = 18, 2 female) groups (Table 1). Children with ASD received their diagnosis using the Autism Diagnostic Observation Schedule (ADOS) and the Autism Diagnostic Interview-Revised (ADI-R) under the guidance of trained UC Davis MIND institute clinicians. TD children were screened using the Social Communication Questionnaire (SCQ), with scores below 15 confirming their TD status. In all children, the ABC assessment was used to evaluate aberrant behaviors, including irritability, lethargy, stereotypic behavior, hyperactivity, and inappropriate speech. Furthermore, the Mullen Scales of Early Learning (MSEL) and the Vineland Adaptive Behavior Scales (VABS) assessments were used to gain insights into secondary behaviors and traits. To help determine whether children with ASD who had GI symptoms (ASD^GI^, n = 12) had differences in Tregs compared to children with ASD without GI symptoms (ASD^NoGI^, n = 24), parents or legal guardians also completed the CHARGE GI history (GIH) assessment, a validated tool used in previous publications [13, 15, 29]. The GIH evaluates the current frequency of GI symptoms within the past 3 months, such as abdominal pain, gaseousness/bloating, constipation, diarrhea, difficulty swallowing, blood in stool/vomit, food sensitivity, and vomiting. The frequency of symptoms was scored on a Likert scale, where (0) = never or rarely, (1) = sometimes or frequently, and (2) = always. Demographic and behavioral information can be found in Table 1. This study received approval from the institutional review boards at the University of California, Davis. Written and informed consent was obtained from the legal guardian or parent for all participants before data collection.

### Peripheral blood mononuclear cell (PBMC) and T Cell Isolation

Blood was collected into citrate tubes from study participants and centrifuged at 2100 RPM. The plasma layer was removed, and blood was resuspended in 1X Hanks Balanced Saline Solution (HBSS) (Corning, CA# 21–021-CM). Blood was then carefully layered onto Lymphoprep (Corning, CA# 25–072-CV) and centrifuged at 1700 RPM for 30 minutes. The PBMC layer was removed and washed with 40mL of 1X HBSS. PBMC were then resuspended in 1mL autoMACS Rinsing Solution (Miltenyi, CA# 130-091-222) supplemented with 0.05% BSA and filtered using a 40μm filter into a new tube. PBMC were then washed 40 with 10mL of autoMACS Rinsing Solution twice at 300 × g to remove contaminating platelets. An aliquot of PBMC was kept for analysis. In addition, following manufacturer’s instructions, PBMC were enriched for CD4^+^CD25^+^ Tregs cells using Miltenyi CD4^+^CD25^+^ Regulatory T Cell Isolation kit, human (CA# 130-091-301). Cells derived from this isolation process were then used for flow cytometry and RNA sequencing.

### Cellular Activation and Flow Cytometry of Tregs phenotypes

PBMC and T cells were rested or activated using CD3/CD28 conjugated beads provided by the Miltenyi T cell activation/expansion human kit. (CA# 130-091-441). 24 hours post activation, cells were stained for flow cytometry analysis. Viability was accessed by staining with the Zombie Aqua Fixable Viability kid (BioLegend, CA# 423101) for 15 minutes. Cells were then washed twice with PBMC wash (1% BSA, 1% sodium azide), followed by incubation with Human TrueStain FcX (BioLegend CA# 422302) for 5 minutes. Without removing the Fc block, the following antibodies were added: CD3- BV605(clone UCHT1), CD4-Alexa Flour 700 (clone RPA-T4), CD25-PE, Foxp3-Alexa 488(clone 206D), CD127-APC Fire 750(clone A019D5), a4b7-PercP-Cy5.5(clone FIB504), and BV421 conjugated CCR9(clone L053E8) or CD39(clone A1) or Glycoprotein A repetitions predominant (GARP)(clone 7B11) or cytotoxic T-lymphocyte associated protein 4 (CTLA-4)(clone BNI3) in combination with APC conjugated CD45RA(clone HI100) or TIGIT(clone A15153G) or Glucocorticoid-induced Tumor necrosis factor receptor-related protein (GITR) (clone 108 − 17) or latency-associated peptide (LAP) (clone TW4–2F8)-APC. All antibodies were acquired though BioLegend. Data was read on a BD LSR 2 and data was visualized prior to analysis using FlowJo v9 software.

### Tregs isolation, RNA extraction and sequencing

In a subset of subjects, CD4^+^CD25^+^Tregs were isolated for RNA sequencing (RNAseq) analysis (ASD = 13, 2 female; TD = 9, 1 female). After isolation, Tregs were flash frozen and stored at −80°C until analysis. RNA was isolated from stored samples using the Zymo Research Quick DNA/RNA Miniprep Plus extraction kit (CA# D7003). Flash frozen cells were resuspended in Proteinase K supplemented DNA/RNA Shield and incubated at room temperature for 30 minutes. Samples were then centrifuged and the RNA containing supernatant was removed and resuspended in DNA/RNA Lysis buffer at a 1:1 ratio, before being transferred to spin-away filters and centrifuged at 10,000g for 30 seconds. A 1:1 volume for 95% ethanol was added to the flow through and transferred to a Zymo-Spin IIICG Column and centrifuged. Column contents were treated with DNase I for 15 minutes, followed by a wash with DNA/RNA Prep-Buffer. Columns were washed twice with DNA/RNA Wash buffer and resuspended in 30μl of DNase/RNase-Free Water. RNA quality was assessed an Aligent Bioanalyzer. Samples with a DV200 score greater than 30% were submitted for library preparation. Libraries were prepared for 3’ Tag-Sequencing (RNAseq) (Lexogen) using 20ng of input RNA. Single end 80bp reads were sequenced on an Aviti sequencer.

### Statistical Analysis

Flow cytometry data passed normality tests; therefore, parametric tests were used for downstream analysis. Outlier removal was performed using ROUT, with Q = 1%. Unpaired Student’s t tests were used to access between group comparisons. Spearman correlations were calculated as they are more appropriate for the variability that comes with small sample sizes compared to Pearson correlations [30, 31]. Correlations between cell frequency, behaviors, and GI symptoms were generated. P-values were then corrected for multiple comparisons using the false discovery rate (FDR). FDR corrected p-values < 0.05 were considered statistically significant. Analysis and visualization of flow cytometry data was performed using GraphPad v9 and the SAS program JMP v16.

RNAseq Statistical Analysis data analysis and visualization was performed using the Partek Flow Software (Illumina). Raw data with Phred 33^+^ scores greater than 25 were aligned using the Spliced Transcripts Alignment to a Reference (STAR) local aligner. Samples with less than 50% alignment were removed from analysis. Aligned reads were quantified with an annotation model and the resulting gene counts were used for statistical analysis. Features with counts less than 1 were filtered prior to DESeq2 42 median ration normalization. Differential gene expression (DEGs) statistical analysis was performed using DESeq2 using Diagnosis and GI status as model parameters. Multiple testing was corrected for using FDR. Genes with an FDR corrected p-value < 0.05 were considered significant. DEGs were analyzed and visualized for enriched Gene Ontology (GO) terms and Kyoto Encyclopedia of Genes and Genomes (KEGG) pathways using the online resource Metascape [32]. Multiple testing for GO and KEGG terms was corrected for using Benjamini Hochberg (BH) tests. BH adjusted P values < 0.05 were considered significant

## Results

### Tregs frequencies are lower in ASD children with GI co-morbidities.

**Figure 1:**
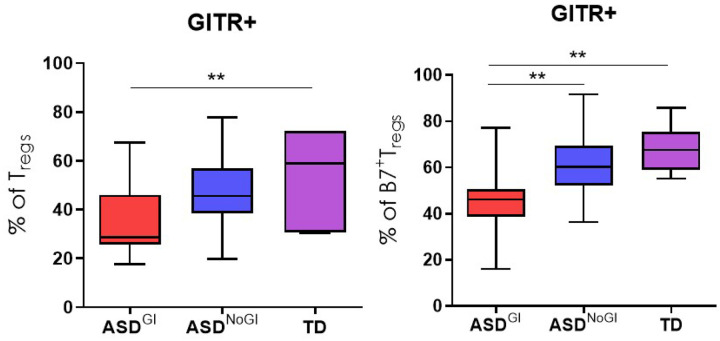
GITR expression on Tregs from ASD children with or without GI symptoms. Flow cytometry was used to access GITR expression on non-gut homing (left) and gut-homing a4b7^+^ Tregs (right) from ASD^GI^, ASD^NoGI^, and TD kids. Both non-gut homing and gut-homing GITR expressing Tregs were significantly lower in ASD^GI^ children compared to TD controls. In gut-homing GITR^+^ Tregs, ASD^GI^ children had significantly lower frequencies than ASD^NoGI^ children also. Significance was determined as p<0.05 (** = p-value <0.005).

**Figure 2:**
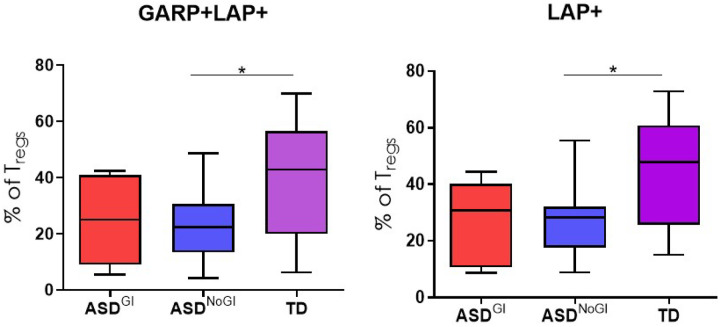
GARP and LAP expression on Tregs in ASD children with or without GI symptoms. Flow cytometry was used to access GARP and LAP expression on Tregs from ASD^GI^, ASD^NoGI^, and TD children. Tregs expressing LAP, as well as those co-expressing GARP and LAP surface markers, were significantly lower in ASD^NoGI^ children compared to TD controls. Significance was determined as p<0.05 (*).

We have previously identified distinct immune profiles in ASD subjects based on the presence of GI issues [18, 33–35]. We were unable to detect statistically significant differences in TIGIT and CTLA-4 Tregs among the groups. CD39^+^ Tregs were reduced in frequency in ASD^GI^ compared to TD controls but did not reach statistical significance after correction for multiple comparisons (Table 2, 3 and 4). When analyzing CD3^+^CD4^+^ that were positive for the gut homing molecule a4b7, ASD^GI^ subjects had lower frequencies of a4b7^+^CD127^−^CD25^+^Foxp3^+^ Tregs compared to ASD^NoGI^ subjects (ASD^GI^ mean = 6.1±1.2; ASD^NoGI^ mean = 7.8±2.5, p = 0.017). ASD^GI^ subjects also had fewer activated GITR^+^CD127^−^CD25^+^Foxp3^+^ Tregs compared to TD controls (ASD^GI^ mean = 34±14.5; TD mean = 54.8, SD = 18.1, p = 0.008) with a trend towards lower frequencies compared to ASD^NoGI^ subjects (mean = 47±16.4, p = 0.059) but this did not reach statistical significance (Figure 1, Table 4). In addition, ASD^GI^ subjects exhibited lower α4β7^+^GITR^+^CD127^−^CD25^+^Foxp3^+^ Tregs (ASD^GI^ mean = 44±15; TD mean = 67.9±10, p = 0.006 compared to the TD and ASD^NoGI^ (ASD^NoGI^ mean = 60.8±13, p = 0.001) (Figure 1, Table 4). Conversely, there were decreased activated LAP^+^CD127^−^CD25^+^Foxp3^+^ Tregs in ASD^NoGI^ (ASD^NoGI^ mean = 29.6±13.6; TD mean = 45.5±20.2, p = 0.04) compared to TD controls and were similar to the ASD^GI^ group (mean = 28± 13.8) (Figure 2). ASD^NoGI^ also had a decrease in GARP^+^ LAP^+^CD127^−^CD25^+^Foxp3^+^ Tregs compared to TD controls (ASD^NoGI^ mean = 23.7±13.5, TD mean = 39.7±22.2, p = 0.05), and again were comparable to ASD^GI^ subjects (mean = 24.6±14.1)(Figure 2). Similar findings also held true for α4β7^+^GARP^+^ CD127^−^CD25^+^Foxp3^+^ Tregs, which were decreased in ASD^NoGI^ compared to controls (ASD^NoGI^ mean = 38.8±15.4, TD mean = 53.5±13.3 p = 0.046), but not ASD^GI^ subjects (mean = 42.5±19.6)). In summary, our data suggests differences in Tregs in ASD compared to TD controls with differences in GITR staining dependent on GI status.

### The RNA transcriptome in isolated Tregs from children with ASD is enriched in genes related to chromatin organization and metabolism

**Figure 3:**
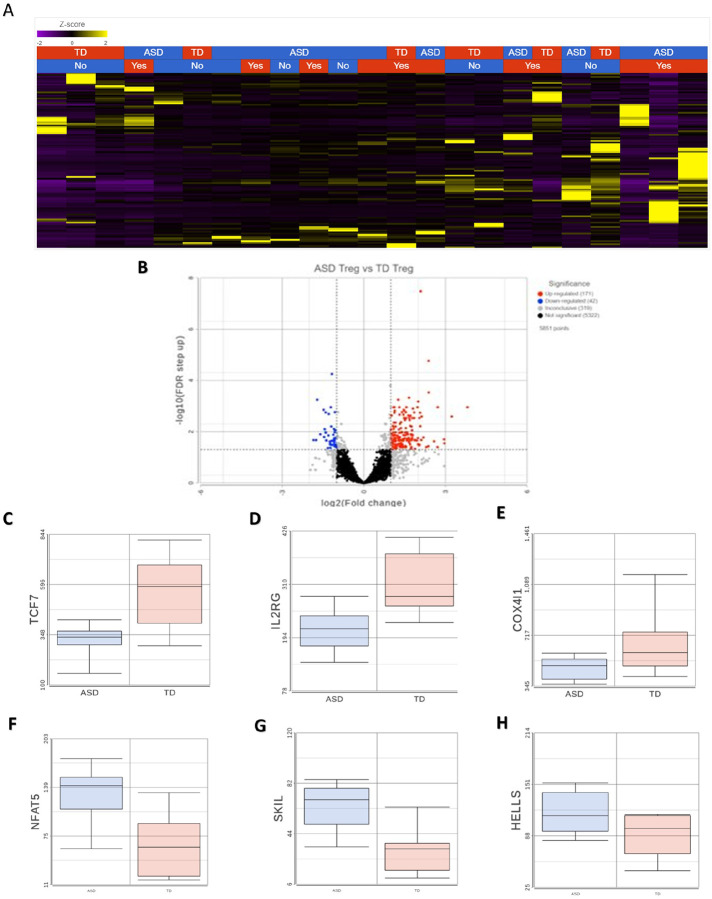
Transcriptional differences in Tregs from ASD and TD children. Bulk RNAseq was used to probe for transcriptional differences between ASD and TD Tregs. **A**Unsupervised hierarchical clustering was used to determine similar groups of subjects, with the first level being diagnosis and second being the presence (“Yes”) or absence (“No) of GI symptoms. Gene expression (rows) is represented by the Z score. **B** A Volcano Plot displaying differentially expressed genes that pass the p-value adjustment threshold. A total of 213 genes were identified as differentially expressed. **C – E** Individual downregulated genes *TCF7* (C), *ILRRG* (D) and *COX41* (E) between diagnostic conditions. **F – H** Individual upregulated genes *NFAT5* (F), *SKIL* (G) and *HELLS* (H) between diagnosis conditions.

**Figure 4:**
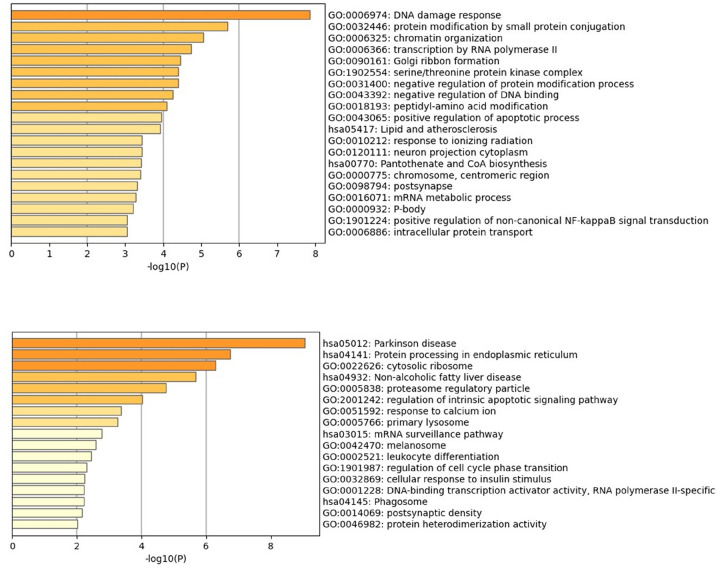
Differentially expressed Tregs genes in ASD are enriched in DNA remolding and metabolic pathways. Gene Ontology (GO) and Kyoto Encyclopedia of Genes and Genomes (KEGG) term enrichment analysis of upregulated (A) and downregulated (B) genes. **A**Upregulated DEGs from ASD Tregs were enriched in multiple GO terms related to DNA remolding, including ‘DNA damage response’ (GO:0006974), and ‘chromatin organization’(GO: 0006325). Significant KEGG pathways included ‘lipid and atherosclerosis’ (hsa05417) and ‘Pantothenate and CoA biosynthesis’ (hsa00770). **B** Downregulated DEGs from ASD Tres were enriched pathways related to metabolism, including ‘Parkinson disease’(hsa05012), as well as immune function (‘leukocyte differentiation’) and protein biology (‘Protein processing in endoplasmic reticulum’).

Unsupervised clustering revealed transcriptional differences between ASD and TD groups (Figure 3A). We identified 213 DEGs when comparing Tregs from children with ASD to TD controls, of which 171 were upregulated and 42 downregulated (Figure 3B). Upregulated genes were enriched in several GO terms related to DNA remodeling, including ‘DNA damage response’, chromatin organization’ and ‘negative regulation of DNA binding’ (Figure 3B). These terms consisted of genes such as the helicase, lymphoid specific (HELLS) (Figure 3H) which is involved in methylation activity, enhancer of zest 1 polycomb repressive complex 2 (EZH1) also involved in the methylation of histones, and the transcription activation suppressor (TASOR) gene. Furthermore, upregulated DEGs were enriched in terms and pathways related to metabolism, including the KEGG pathway ‘lipid and atherosclerosis’, including genes such as low-density lipoprotein receptor (LDLR) and POU Class 2 Homeobox 1 (POU2F1), and the ‘Panthothenate and CoA biosynthesis’ pathway, including the genes aldehyde dehydrogenase 3 family member A2 (ALDH3A2) and pantothenate kinase 3 (PANK3) (Figure 4A). There were several upregulated genes associated with immune signaling, such as mitogen-activated protein kinase 3 (MAPK3), janus kinase 2 (JAK2), nuclear factor of activated T cells 5 (NFAT5) (Figure 3F), SKI like proto-oncogene (SKIL) (Figure 4) and zinc finger MIZ-type containing 1 (ZMIZ1). For the 43 downregulated genes, these were enriched for terms related to processes involved in oxidative phosphorylation, such as the GO Biological process terms ‘cellular respiration’ and ‘oxidative phosphorylation’ (Figure 4B, Supplementary File 2). In addition, other terms related to immune function, including ‘MAPK signaling pathway’ and ‘leukocyte differentiation’, and included the transcription factor 7 (TCF7) (Figure 3C) and the interleukin 2 receptor subunit gamma (IL2RG) gene (Figure 3D, Supplementary File 2). KEGG pathways were associated with protein processing and protein localization, such as the ‘protein processing in endoplasmic reticulum’ pathways (Supplementary File 2). An enrichment of terms related to the cellular stress response was found, including the GO term ‘regulation of intrinsic apoptotic signaling pathway’, which included the genes endoplasmic reticulum to nucleus signaling 1 (ERN1), selenoprotein S (SELENOS), transcription factor AP4 (TFAP4) and cell cycle and apoptosis regulator 2 (CCAR2). These data suggest that there are dysregulated metabolic and DNA remodeling programs in Tregs from ASD, in addition to an altered stress state.

Next, we considered how GI status may influence DEG enrichment. Comparison of mRNA data from ASD^GI^ and TD Tregs reveal a total of 19 DEGs, with 4 upregulated and 15 downregulated (Supplemental File 1). Upregulated genes were associated with the meiotic cell cycle (PDIK1L) and tumor proliferation (BACH1 and PDIK1L). Within the downregulated genes, several were involved in mitochondrial function (MT-ND1, MT-CYB, MT-ATP6, and CYB5R1). In ASD^NoGI^ and TD comparisons, only 7 DEGs were observed (5 downregulated/2 upregulated) after multiple comparison adjustment (Supplemental File 1). Upregulated genes consisted of MIB2 and SLC39A10 and did not seem to be involved in any pathway. Downregulated genes included DPP7, CIRBP, MT-ND1, RBM3 and TRAPPC6A and did not appear to be involved in any molecular, cellular or biological pathway.

### Fewer Tregs are associated with worse behaviors in children with ASD and TD controls.

**Figure 5:**
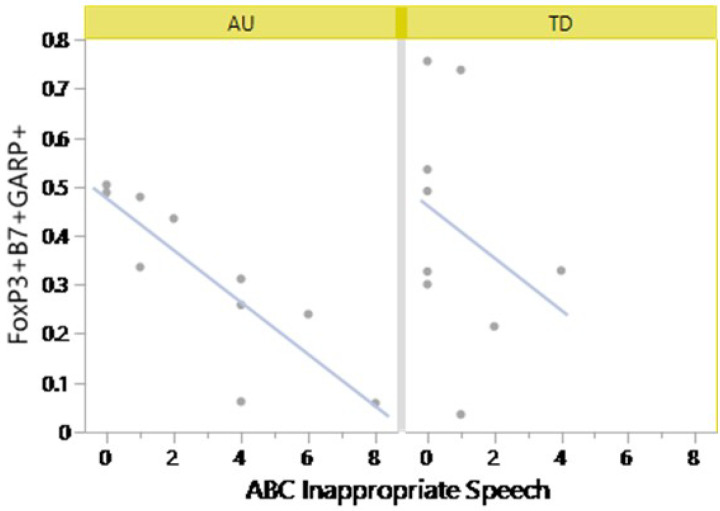
Gut homing GARP^+^CD127^−^CD25^+^Foxp3^+^ Tregs are associated with improved behaviors. Spearman Rho correlations were used to access associations between baseline and activated Tregs frequencies and ADI, MSEL, VABS and ABC assessment scores in ASD and TD subjects. Significant correlations were observed between gut-homing a4b7^+^GARP+CD127^−^CD25^+^Foxp3^+^ Tregs and Inappropriate Speech behavior within the ASD^NoGI^ group (rho = −0.9384, FDR P-value = 0.0021).

To gain further insights into the relationship between Tregs and ASD related behaviors, we investigated whether there were associations between behavioral scores, the GI assessments and the phenotypes of Tregs (Table 5). In the context of ASD, we identified a significant association between increased a4b7^+^GARP^+^CD127^−^CD25^+^Foxp3^+^Tregs and improved ABC inappropriate speech scores in the ASD^NoGI^ group. (rho = −0.9384, FDR p-value = 0.0021) (Figure 5). However, after multiple comparisons correction, no significant correlations were observed in the ASD^GI^ group alone or based on GIH assessment scores across the ASD groups. In the TD group, decreased Tregs were associated with lower adaptive behaviors, primarily between GARP^+^LAP^+^CD127^−^CD25^+^Foxp3^+^ and LAP^+^CD127^−^CD25^+^Foxp3^+^ Tregs that were associated with reduced VABS composite (rho = −0.833, FDR p-value = 0.016) and daily living scores (rho = −0.7833, FDR p-value = 0.037), respectively.

## Discussion

Immune dysregulation is a common feature observed in children with ASD. Immune suppression via Tregs is essential for maintaining homeostasis. Disruptions to regulatory mechanisms can result in inflammation and an increased likelihood of developing immune related conditions. In the current study, we sought to characterize Tregs in ASD. When children with ASD were stratified based on the presence of GI symptoms, we found that ASD^GI^ children had reduced GITR^+^CD127^−^CD25^+^Foxp3^+^Tregs compared to TD controls, and reduced gut homing a4b7^+^GITR^+^CD127^−^CD25^+^Foxp3^+^Tregs compared to ASD^NoGI^ and TD controls. Furthermore, we observed lower LAP^+^CD127^−^CD25^+^Foxp3^+^Tregs, GARP^+^ LAP^+^CD127^−^CD25^+^Foxp3^+^ Tregs, and gut homing α4β7^+^GARP^+^CD127^−^CD25^+^Foxp3^+^ Tregs in ASD^NOGI^ compared to their TD counterparts. Analysis of gene expression in isolated CD4^+^CD25^+^Tregs revealed that upregulated genes in children with ASD were primarily involved in DNA remodeling/modification, metabolism and immune signaling. Downregulated genes in ASD belonged to processes involved in oxidative phosphorylation, as well as genes involved in T cell differentiation and protein biology. This data suggests that Tregs subpopulations are altered in ASD children and that different frequencies of subphenotypes of Tregs were altered dependent on whether GI issues are present. Moreover, we find evidence suggesting that DNA repair mechanisms and DNA accessibility are altered in Tregs isolated from children with ASD, possibly driven by changes in cellular metabolism. Finally, we found associations between worse behaviors and decreased Tregs both in the context of ASD and TD. Restoring Tregs function may thus prove to be a beneficial strategy for ameliorating potentially harmful immune activation and ASD related behaviors.

Inflammation in peripheral, central nervous system, and GI tissues is frequently observed in ASD. Increased peripheral levels of pro-inflammatory cytokines IL-1β, IL-6, IL-8 and IL-12p40 are seen in ASD and associated with more impaired behaviors [36]. Elevated levels of TNFa, IL-6, IFNg, GM-CSF and IL-8 are also observed in the post-mortem brain tissue of ASD children [37]. Evidence supporting inflammatory processes in the GI tissue of children with ASD has also been documented [38]. These findings may in part be due to the increased frequencies of activated immune cells in each of these respective tissues [17, 22, 39, 40]. However, another contributing factor to this seemingly systematic inflammation is dysregulated Tregs biology. In ASD, lower frequencies of peripheral Tregs and lower levels of canonical Tregs-derived cytokines such as TGFb, IL-10 and IL-35 are reported [21, 41–43]. Deficiencies in putative Tregs were first identified by Warren et al., over 35 years ago, who identified lower peripheral frequencies of CD4^+^CD45RA^+^ T cells [44]. Ahmed and colleagues found that transcription factors associated with a Tregs phenotypic identity, primarily Foxp3 and Helios transcription factors, are reduced in ASD; however, so far, few studies have attempted to identify different Treg phenotypes in ASD [45, 46]. Using CD127^−^CD25^+^Foxp3^+^ as the basis to identify Tregs, the results of the current study show that various Tregs phenotypes may be lower in children with ASD. The frequencies of Tregs that were positive for the anti-inflammatory molecules LAP^+^, GARP^+^, and GITR^+^ were different in ASD compared to controls.

The immune modulatory marker LAP is non-covalently associated with active TGFb1, rendering TGFb1 biologically inactive until it is cleaved [47]. GARP can then bind to LAP and upon tethering to the Tregs surface, TGFb1 is converted to its activated form though integrins, resulting in the release of active TGFβ1 [47, 48]. In the present study, we find evidence of GARP and LAP dysregulation in ASD children. We observe lower frequencies of LAP^+^GARP^+^CD127^−^CD25^+^Foxp3^+^ and LAP^+^CD127^−^CD25^+^Foxp3^+^ Tregs, as well as reduced gut homing a4b7^+^GARP^+^CD127^−^CD25^+^ Foxp3^+^ Tregs. There are several possible reasons as to why there are decreased GARP^+^ and LAP^+^ Tregs, such as genetic mutations, inefficient Tregs activation, or dysregulated membrane trafficking [49, 50]. For example, in a study by Lehmkuhl et. al., investigating patients with primary immunodeficiencies, mutations in the GARP gene, LRRC32, was associated with severely reduced Tregs populations and reduced suppressive activity [49]. Furthermore, reduced GARP expression can contribute to a destabilized Tregs phenotype. Mechanistically, this may result from GARP mediated regulation of the histone deacetylase HDAC9. A deficiency in GARP increases Hdac9 activity and deacetylates Foxp3 (the Tregs “master controller”) at a greater rate, leaving it more susceptible to removal from the cellular system [49]. Reduced LAP may be a consequence of reduced GARP expression, as the latter tethers the former to the cell surface [51]. Of note, in this study, we found that reduced GARP^+^ and LAP^+^ Tregs are associated with worse inappropriate speech scores. GITR is a member of the TNF-receptor superfamily and binds to its ligand, GITRL, found on antigen presenting and endothelial cells [54]. Tregs express high levels of GITR on their membranes, where it acts as a co-stimulatory molecule [55]. GITR^+^ Tregs are markedly lower in frequency in autoimmune diseases including type 1 diabetes and systemic lupus erythematous, and increasing the frequency of GITR^+^ Tregs is associated with the remission in these conditions [56–59]. The role of GITR signaling in Tregs is nuanced, with data supporting GITR signaling in Tregs proliferation, expansion and prevention of inflammatory colitis in mice, while others have suggested that during GITR mediated proliferation suppressive activity is abrogated [56, 58, 59]. Possibly as a mechanism to increase cell division and Tregs numbers at sites of inflammation before function is fully restored. In the present study, we observe reduced frequencies of GITR^+^CD127^−^CD25^+^ Foxp3^+^Tregs in ASD^GI^ children. The function and significance of GITR^+^ Tregs in ASD children is unknown, although it can be speculated that reduced Tregs may lead to GI related issues as shown in mice models [58, 59].

To the best of our knowledge, the RNA transcriptome of isolated Tregs in the context of ASD has not been investigated. In our study, we identified several biological pathways that are crucial for Tregs mediated suppression and for shaping Tregs identity. Firstly, many of the observed upregulated DEGs were involved in DNA remolding, such as HELLS and EZH1, both of which are involved in DNA methylation. Other significantly enriched GO terms included ‘chromatin organization’, consisting of genes involved in transcriptional regulation (Transcription Activation Suppressor; TASOR, Tousled-like kinase 1; TSK1, Myb Like, SWIRM and MPN Domains 1; MYSM1) as well as genes associated with histone deacetylases (ring finger protein, LIM domain interacting; RLIM, ligand dependent nuclear receptor interacting factor 1; LRIF1) [60–63]. Reorganization of chromatin structures and methylation at promoter regions of Tregs signature genes is essential for the development and maintenance of Tregs, with some subpopulations more resistant to changes in epigenetic status than others. Demethylation at the Treg-specific demethylated region (TSDR) within the Foxp3 locus is considered essential for Tregs development [64]. Upregulated expression of genes involved in epigenetic processes may indicate that epigenetic imprints at essential sites for Tregs phenotype stability are altered and may also contribute to reduced populations in ASD. Moreover, genes that negatively regulate Foxp3 activity were upregulated, such as those involved in ubiquitination, including E3 ubiquitin ligases (HECT, UBA and WWE domain containing 1, E3 ubiquitin protein ligase; HUWE1, mind bomb 1; MIB1), and those that target ubiquitin tagged proteins for autophagy (sequestosome-1, SQSTM1). Foxp3 activity is in part regulated via protein modifications such as ubiquitination and acetylation [65]. While both work to negatively regulate Foxp3 function, acetylation is reversable whereas ubiquitin tags molecules for permanent degradation. These changes in DNA remodeling machinery and protein modification systems could contribute to dysregulated Tregs phenotypes and consequently lower peripheral Tregs populations in ASD and warrant further investigation.

Dysregulated Tregs may be due to altered cellular metabolic programs. Many of the metabolites and cofactors produced from cellular metabolism modulate processes that control Tregs development and function [66]. Activated Tregs are associated with increased glycolytic demands for energy and are tightly regulated to modulate Tregs phenotype and function [66, 67]. For example, fatty acid metabolism and subsequent oxidative phosphorylation is associated with superior suppressive capabilities in Tregs [66]. In the present study, we identify several dysregulated metabolic genes. Upregulated genes were involved in aspects of lipid metabolism, including low-density lipoprotein receptor (LDLR), salt inducible kinase 2 (SIK2) – involved in fatty acid oxidation, the zinc finger DHHC-type palmitoyltransferase 21 (ZDHHC21) – a hormone receptor, and pantothenate kinase 3 (PANK3) 51 gene - involved in Coenzyme A synthesis. Furthermore, GO terms associated with amino acid metabolism, such as ‘serine family amino acid metabolic process’ were found. Conversely, downregulated genes suggest disruptions in mitochondrial respiration and oxidative phosphorylation (OXPHOS). Significantly disrupted GO Biological Process terms included ‘electron transfer activity’, ‘aerobic respiration’ and ‘oxidative phosphorylation’ (Supplemental data). Genes within these processes included those involved with mitochondrial cytochromes (cytochrome C oxidase subunit 4I1; COX4I1, cytochrome B; CYTB) and mitochondrial complex 1 (NADH dehydrogenase 1; ND1, NADH:upiquinone oxidoreductase subunit A10;NDUFA10). These data suggest that there are differences in Tregs metabolism between ASD and TD children, which could subsequently impact their phenotype and function. A recent single-cell RNA sequencing study evaluated metabolic differences in Tregs showed amino acid metabolism, specifically polyamine metabolism, was downregulated in Tregs [68]. Our studies suggest that ASD Tregs may preferentially undergo beta-oxidation and that genes involved with downstream tricarboxylic acid (TCA) and OXPHOS activity are dysregulated.

There are several limitations to our study that require addressing in future studies. As FoxP3 is X linked, females may have different phenotypes and gene expression of Tregs compared to males. This study was not powered to determine differences in Tregs populations and gene expression changes between male and female groups based on diagnosis. Furthermore, due to the absence of a TD^GI^ group, we were unable to compare ASD^GI^ and TD^GI^ groups in our study. This has been an issue in prior studies due to the rarity of TD with GI issues in this age group [13, 15]. Furthermore, a large variety of cells with regulatory function have been described and we initially focused on some of the better described surface molecules. Further studies can expand the phenotypes assessed. We were also limited by the number of cells that could be collected from each pediatric blood sample. Lastly, we did not identify differences in the transcriptome of Tregs between ASD^NoGI^ and ASD^GI^ groups, which may be due to sample size. Despite these limitations, we feel that the information provided in the present study significantly advances the understanding of Tregs dysregulation in ASD and points towards the need for further studies in this area.

Tregs dysregulation may be at the root of many inflammatory processes that occur in ASD. In this study, we identified altered Tregs phenotypes in ASD, with some phenotypes being dependent on the presence of GI symptoms. We have also found that the gene pathways in Tregs are dysregulated in ASD. These pathways may affect DNA repair and cellular metabolism in ASD. Lastly, we showed that decreased Tregs populations are associated with worse behaviors. Taken together, these data confirm the presence of Tregs dysregulation in ASD. Further evaluation of changes in Tregs metabolism and function in ASD are warranted. The therapeutic potential for drugs involved with metabolism that could be repurposed for increasing immunoregulatory function (Metformin) have already been identified in clinical trials for neurodevelopmental disorders and preclinical models of altered neurodevelopment [69, 70]. Continued efforts are needed to understand the relationship between Tregs and ASD.

## Supplementary Material

Supplementary Files

This is a list of supplementary files associated with this preprint. Click to download.

- ChargebackTregFlowPaperTablesmeanSD11092025.docx
- CopyofCopyofTregmRNAandFlowAnalysisSupplementalFile1.xlsx
- CopyofCopyofTregmRNAandFlowAnalysisSupplementalFile2.xlsx

## Data Availability

The datasets used for the current states are available from the corresponding author upon reasonable request.
